# Immunoglobulin light chain amyloidosis diagnosis and treatment algorithm 2021

**DOI:** 10.1038/s41408-021-00483-7

**Published:** 2021-05-15

**Authors:** M. Hasib Sidiqi, Morie A. Gertz

**Affiliations:** 1 Haematology Department, Fiona Stanley Hospital, Perth, WA Australia; 2 Division of Hematology, Department of Internal Medicine, Mayo Clinic, Rochester, MN USA

**Keywords:** Myeloma, Chemotherapy

## Abstract

Immunoglobulin light chain amyloidosis (AL) commonly presents with nephrotic range proteinuria, heart failure with preserved ejection fraction, nondiabetic peripheral neuropathy, unexplained hepatomegaly or diarrhea, and should be considered in patients presenting with these symptoms. More importantly, patients being monitored for smoldering multiple myeloma and a monoclonal gammopathy of undetermined significance (MGUS) are at risk for developing AL amyloidosis. MGUS and myeloma patients that have atypical features, including unexplained weight loss; lower extremity edema, early satiety, and dyspnea on exertion should be considered at risk for light chain amyloidosis. Overlooking the diagnosis of light chain amyloidosis leading to therapy delay is common, and it represents an error of diagnostic consideration. Herein we provide a review of established and investigational treatments for patients with AL amyloidosis and provide algorithms for workup and management of these patients.

## Introduction

The incidence of AL amyloidosis is estimated to be three to five patients per million per year^1^. This statistic would make it approximately one-fifth as common as multiple myeloma^2^. In the United Kingdom, the incidence is ~1 per 100,000^3^. The Medicare claims database suggests that the mean age of AL amyloidosis at diagnosis is 63 with an incidence of 10–14 patients per million per year with a prevalence higher in males^4^. Wild-type transthyretin (TTR) amyloidosis is increasingly being recognized and may be present in a quarter of the elderly at postmortem and is seen in 13–19% of patients with heart failure and preserved ejection fraction, likely making it the most common form of systemic amyloidosis^5^.

The diagnosis of AL amyloidosis should be considered by physicians in any patient seen with nephrotic range proteinuria, heart failure with preserved ejection fraction^6^, nondiabetic peripheral neuropathy^7^, unexplained hepatomegaly^8^, or diarrhea. However these signs and symptoms are not specific to amyloidosis. Heart failure with preserved ejection fraction, one of the most common manifestations of AL amyloidosis, can be misdiagnosed because the echocardiogram has nonspecific findings. Wall thickening can be misinterpreted as hypertension with hypertrophy or hypertrophic cardiomyopathy^9^. Cardiac magnetic resonance imaging with gadolinium is a more specific test, however is not a screening test and often only ordered if amyloidosis is suspected^10^. A pseudoinfarction pattern seen on the EKG could be misinterpreted as true ischemic disease. Patients with peripheral neuropathy and a monoclonal gammopathy are frequently misdiagnosed as CIDP (chronic inflammatory demyelinating polyneuropathy)^11^. Patients often undergo unnecessary therapies such as immunoglobulin infusions and plasma exchange for many months prior to evaluation for AL amyloidosis. Other clinical features of AL amyloidosis such as tongue enlargement or periorbital purpura, whilst highly specific, are only found in 15% of patients and are not effective screening tools for AL amyloidosis.

For the cancer provider following patients with monoclonal gammopathy of undetermined significance (MGUS) or smoldering multiple myeloma, it is important to keep in mind that these patients are not monitored solely for the development of myeloma. Some develop lymphoma or Waldenström macroglobulinemia, and a small percentage develop light chain amyloidosis^12^. At Mayo Clinic, 9% of all patients seen with a monoclonal gammopathy are ultimately proven to have light chain amyloidosis. Even adjusting for referral bias, 3–4% of all patients with monoclonal proteins have light chain amyloidosis. Furthermore, if a provider does not see one patient with AL amyloidosis for every five patients with multiple myeloma, it is likely the diagnosis is being overlooked^13^.

The ongoing rates of early mortality in newly diagnosed AL amyloidosis suggest there continue to be significant delays in diagnosis^14^. Nearly 20% of patients succumb to the disease within 6 months of diagnosis, and this statistic has shown no improvement in 40 years, suggesting, that patients who are diagnosed at an advanced state cannot be helped despite major advances in therapy for this disease.

An online survey from the Amyloid Research Consortium indicates that 37% of patients are diagnosed over 1 year from the onset of initial symptoms with a median of three physician visits before a diagnosis is established. Cancer care providers constitute 34% of the specialists that are diagnosing this disorder, far greater than nephrologists, cardiologists, and gastroenterologists^15^.

**Fig. 1:**
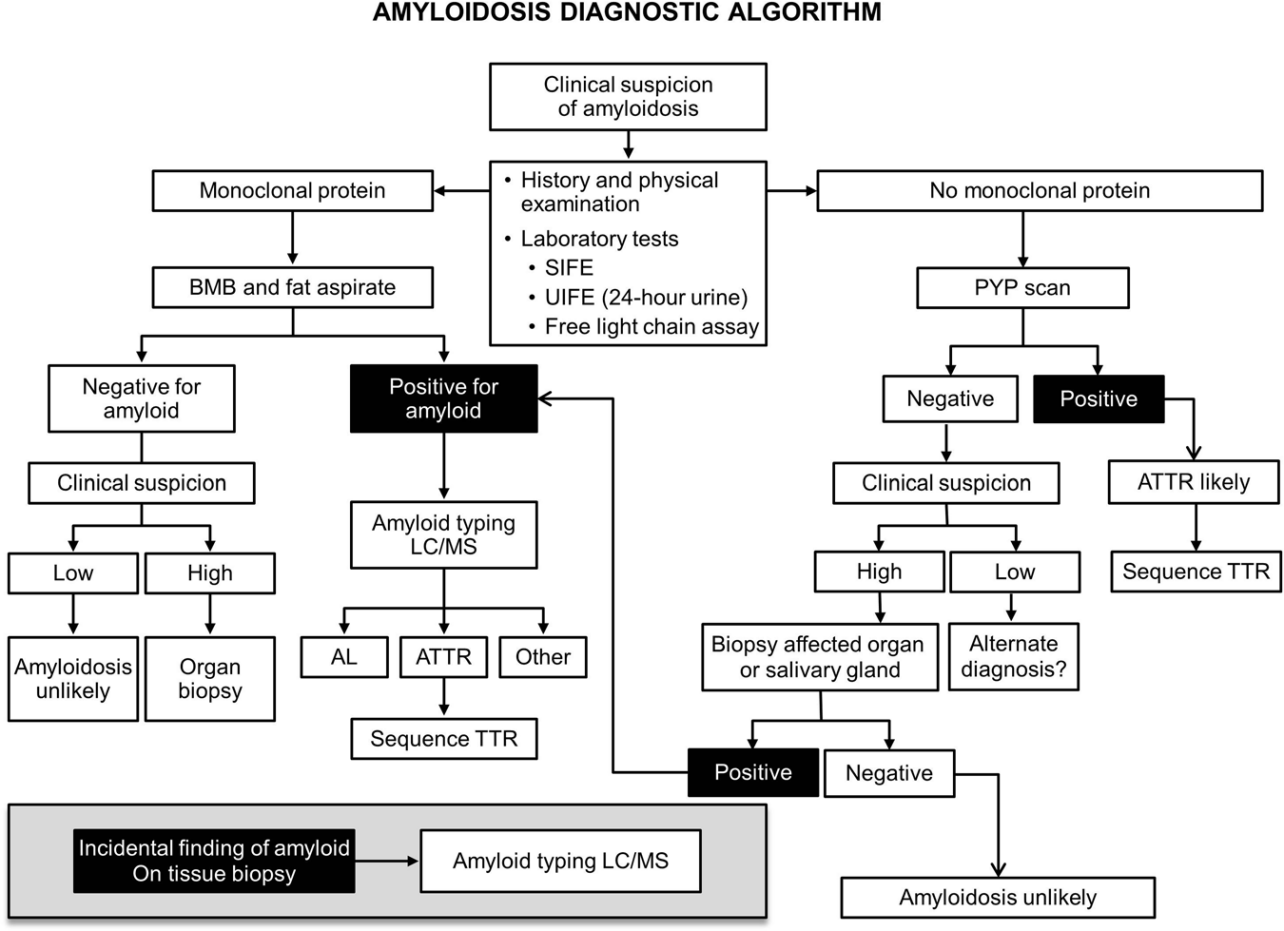
Diagnostic algorithm for patients that are being evaluated for a syndrome compatible with systemic amyloidosis. ATTR transthyretin amyloidosis, AL immunoglobulin light chain amyloidosis, TTR transthyretin, BMB bone marrow biopsy, SIFE serum immunofixation electrophoresis, UIFE urine immunofixation electrophoresis, LC/MS liquid chromatography-coupled tandem mass spectrometry, PYP (99 m)Tc-pyrophosphate scintigraphy. Figure reproduced from “Two types of amyloid in a single patient: a case series”.

If AL amyloidosis is suspected, particularly in patients who have multi-organ dysfunction, biopsies are not the first step in screening. Currently, 71% of patients that are seen have cardiac involvement, 58% have renal involvement, 23% have nerve involvement, and 16% have liver involvement. Despite these numbers, the majority of patients with cardiac, renal, hepatic, and nerve problems will not have AL amyloidosis. The first screening test for these patients, as shown in the algorithm (Fig. 1), would be serum immunofixation and an immunoglobulin free light chain assay for k and λ immunoglobulin light chains^16^. If the patient has cardiac dysfunction, a pyrophosphate scan, which should be available in most hospitals in the developed world, should also be performed^17^. If these screening tests are positive, the diagnostic pathway is clear. A simple subcutaneous fat aspirate (https://www.youtube.com/watch?v=tctYTmxd9gQ) and a bone marrow biopsy will demonstrate amyloid deposits in over 85% of patients with immunoglobulin light chain abnormalities and confirm a diagnosis of AL amyloidosis. If the bone marrow biopsy and fat aspirate are negative and only if the index of suspicion is high would the patient next move to a direct organ biopsy of the heart, liver, kidney, nerve, etc. Patients with echocardiographic evidence suggesting cardiac amyloidosis, usually demonstrate thickened ventricular walls, Doppler evidence of poor diastolic filling and abnormal longitudinal strain. These patients need to undergo a pyrophosphate scan. A positive scan in the absence of a monoclonal protein in the serum and urine is sufficient for the diagnosis of cardiac TTR amyloidosis^18^. These patients should be referred to a cardiologist for further investigation and management. However distinguishing TTR amyloidosis and AL amyloidosis can be difficult given the increased prevalence of MGUS in patients with TTR amyloidosis and the rare phenomena of two types of amyloidosis presenting concurrently, making subtyping of the amyloid deposits critical^19–22^.

### Classification of amyloid

Accurate typing of the protein subunit responsible for amyloid deposition is important since it directs treatment. This can range from observation to intensive treatment with chemotherapy as is in the cases in systemic AL amyloidosis, making subtyping critical for appropriate management. Historically, immunohistochemistry^23,24^ and immunofluorescence^25^ techniques have been used to subtype the amyloid deposits. However these techniques have limitations. In light chain amyloidosis, commercial antisera purchased to detect κ and λ immunoglobulin light chains are usually directed against epitopes on the constant region of the immunoglobulin light chain. When amyloid light chains are deposited in tissues, usually only a fragment of the intact light chain is deposited, typically the variable portion (V_L_) with a molecular weight of ~12 kd. A normal intact light chain has a molecular weight of 25 kd, suggesting there has been deletion of the constant portion of the light chain, making the immunoglobulin fragment unrecognizable to commercial antisera. Secondly, the light chains in amyloidosis are known to misfold, and the potential of a previously exposed epitope no longer being accessible to the commercial antibody exists, rendering it unidentifiable^26^. Finally, there are at least 30 different types of amyloid proteins, and few centers are equipped with such a large panel of antisera, making it next to impossible to identify rare forms of amyloidosis, such as fibrinogen, LECT2, apolipoprotein, and lysozyme.

The gold standard for typing is laser capture mass spectroscopic proteome analysis^27^. Amyloid deposits are directly micro dissected from a glass slide, and the technique can be performed on archived paraffin-embedded tissues. Peptides are sequenced by a mass spectrometer and then compared with libraries of proteins for identification^28,29^. Although expensive and not available in all laboratories, proteomic analysis with mass spectroscopy remains the gold standard for identification of the amyloid protein subunit.

In a review of over 4000 proteomic analyses of amyloid deposits, 62% were of immunoglobulin origin. However, a full 38% were not of immunoglobulin origin, and chemotherapy would have been contraindicated. Non-immunoglobulin forms of amyloidosis included AA amyloidosis, ALECT2, A-insulin, A-fibrinogen, and A-gelsolin (Kurtin P. personal communication)^30–33^.

### Patient is referred from a specialist with biopsy-proven amyloid

Many specialists, when encountering a patient with biopsy of an organ containing amyloid, refer to a cancer care provider uncertain of the type of amyloidosis. The first step for all biopsied tissues, would be mass spectroscopic analysis (Fig. 1). In patients with AL amyloidosis, measurement of bone marrow plasma cells^34^ and FISH genetics^35^, as would be done in multiple myeloma patients, are indicated. To accurately stage AL amyloidosis testing for NT-proBNP, troponin, and the difference between the involved and uninvolved immunoglobulin free light chain is needed^16^. Cardiac imaging either with echocardiography or magnetic resonance imaging can further rdelineate degree of cardiac involvement, critical for prognosis^36^. For patients with light chain amyloidosis in the absence of symptoms, the role of routine skeletal imaging, as is done in multiple myeloma, is not well defined due to a lack of high-quality evidence.

If TTR amyloid is identified by mass spectroscopic analysis, clinically these patients should have presented with peripheral neuropathy or cardiomyopathy. For patients with cardiac symptoms a pyrophosphate scan of the heart should be performed and if strongly positive would suggest that the amyloid is of TTR origin^37^. Any patient with TTR amyloidosis should have gene sequencing of the TTR gene to distinguish wild-type TTR, as is seen in senile cardiac amyloidosis, from the very rare mutations of TTR that lead to inherited amyloidosis^38^. The genetic mutations causing TTR amyloidosis vary among ethnicities with the most common V30M mutation having clusters in Portugal, Japan and Sweden, whilst others are less common such as the T60A mutation seen in Ireland. Since familial amyloidosis is not treated with chemotherapy, these patients should be referred for genetic counseling, consideration of liver transplant, diflunisal^39,40^ or doxycycline therapy^41^, or one of the agents that suppress translation of liver TTR messenger RNA^42,43^ into the fully-formed TTR protein. Patients with wild-type TTR amyloidosis are usually over the age of 70, 90% are men, and half have carpal tunnel syndrome^44^. Tafamidis, an agent that stabilizes the TTR proteins, is approved therapy for TTR cardiac amyloidosis.

Staging of AL amyloidosis is based on a four-point system where one point is assigned for a DFLC > 18 mg/dL, a cardiac troponin *T* > 0.025 mcg/L, or an NT-proBNP ≥1800 ng/L. This provides a staging system of I, II, III, and IV based on the number of points assigned (0, 1, 2, or 3). The staging system has been validated in multiple datasets, including patients treated with stem cell transplantation, patients on clinical trials, and non-transplant patients treated with standard chemotherapy^45^. Other effective staging systems include a European staging system where Mayo 2004 stage 3 was subclassified into three substages using systolic blood pressure and NT-proBNP at 100 mmHg^46^ and 8500 ng/mL^47^, respectively.

#### Therapy of amyloidosis

**Fig. 2:**
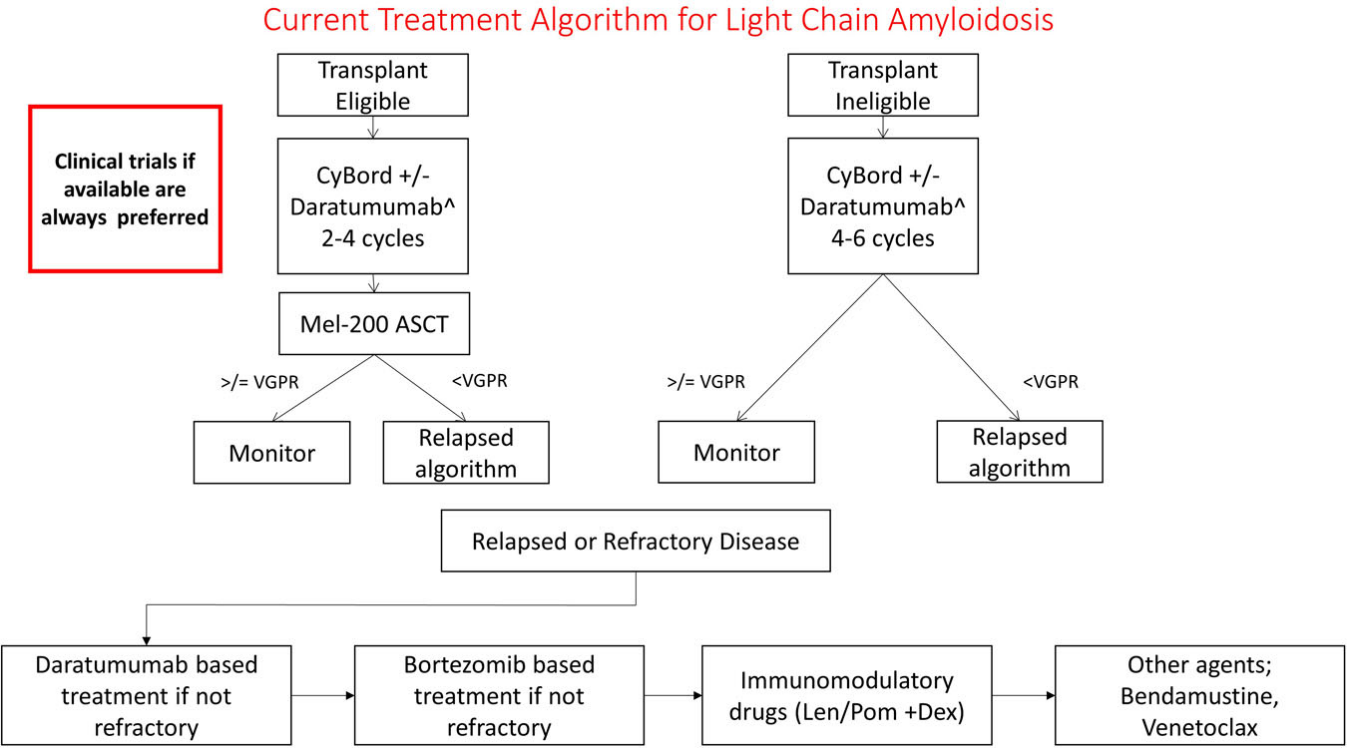
Current treatment algorithm in light chain amyloidosis. CybBord cyclophosphamide, bortezomib, and dexamethasone, ASCT autologous stem cell transplant, VGPR very good partial response, Len lenalidomide, Pom pomalidomide, Dex dexamethasone. ^Where available induction with daratumumab plus CyBord is recommended. If unavailable CyBord is induction is recommended. *Daratumumab base treatment is preferred at first relapse if patient is previously unexposed to this agent.

The first successful treatment for AL amyloidosis was melphalan and prednisone introduced in 1972^48^. Subsequently autologous stem cell transplantation was reported in 1996^49^, and high-dose dexamethasone in 1997^50^. Melphalan and dexamethasone combination therapy was reported in 2004^51^. With the introduction of novel agents for treatment of myeloma, many of these were subsequently utilized in AL amyloidosis including immunomodulatory drugs (IMIDs) such as thalidomide^52,53^, lenalidomide^54,55^, and pomalidomide^56,57^, as well as combinations of IMIDs with alkylating agents^58^. However IMIDs are poorly tolerated in patients, particularly those with cardiac AL amyloidosis^59,60^. The first step in assessing therapy for a patient with AL amyloidosis, as shown in our algorithm (Fig. 2), is determination of their eligibility for stem cell transplantation. Using transplantation in AL amyloidosis is theoretically better than it is for multiple myeloma. Unlike multiple myeloma, the tumor mass being treated is less with a median of ~10% plasma cells at diagnosis and a median dFLC of only 24 mg/dL. Unfavorable genetics, seen in nearly a quarter of patients with multiple myeloma [such as 1q+, t(4;14), and −17p] are present in <5% of patients with light chain amyloidosis. The proliferative rate of plasma cells is lower in AL amyloidosis patients, suggesting that once a response is obtained, it is likely to be more durable than is seen in multiple myeloma^61^. In fact, in the pre-novel agent era, 10-year survival of patients with AL amyloidosis undergoing stem cell transplantation was 43%^62^. A prospective randomized trial of melphalan and dexamethasone with stem cell transplant also favored stem cell transplantation, although the comparator arm did not contain novel agents^63^. With careful patient selection, the therapy-related mortality has been reduced to ~2%^64,65^. Patients that do not achieve greater than a VGPR can have bortezomib-based consolidation posttransplant, which significantly upgrades treatment response posttransplant^66^. A prospective randomized trial demonstrated an improved survival outcome with bortezomib–dexamethasone prior to stem cell transplant^67^. Our current approach is to manage all transplant eligible patients with induction therapy followed by stem cell collection and transplant (Fig. 2).

However it is important to note that no more than 25% of newly diagnosed patients are eligible by virtue of age, renal function, and extent of cardiac failure. The remaining 75–80% are candidates for chemotherapy. Melphalan and dexamethasone demonstrates impressive survival in patients that are capable of receiving full-dose therapy with a median survival of just <8 years^68^. There have been reports of cyclophosphamide–thalidomide–dexamethasone^69^, lenalidomide–dexamethasone, melphalan–dexamethasone–lenalidomide, cyclophosphamide–lenalidomide–dexamethasone^70^, but none of these are currently used in the Mayo Clinic algorithm due to toxicity and the preference for bortezomib. It should be noted that lenalidomide raises the NT-proBNP in AL patients^71^. CyBorD or VCD (cyclophosphamide–bortezomib–dexamethasone) was first reported to be effective in 2012. In the original iteration, cyclophosphamide was given orally weekly, dexamethasone orally weekly, and bortezomib subcutaneously weekly. In this original trial, 17 patients were treated, 10 with symptomatic cardiac involvement with a 94% response rate, and 71% complete response rate with an additional three patients who were previously deemed ineligible for stem cell transplant to become eligible^72^. These results were validated in over 230 patients with AL amyloidosis, demonstrating a median survival in excess of six years, with all patients surviving in stage 1 disease and a median survival of <1 year in stage 4 disease^47^. Survival was dependent on response depth, with patients achieving a VGPR or better having the best outcome. Achievement of a VGPR is used in the algorithm to determine whether second-line therapy should be considered. The utility of frontline bortezomib therapy in AL amyloidosis is further highlighted by a phase III randomized open-label trial comparing bortezomib, melphalan, and dexamethasone with melphalan and dexamethasone in newly diagnosed patients. Response rates were higher in the bortezomib containing arm (79 vs. 52%) as was the rate of VGPR or CR (64 vs. 395%)^73^. Overall survival was improved in the bortezomib arm with a two fold reduction in mortality rate. The UK national Amyloidosis center reported a prospective observational study of 915 patients treated with frontline bortezomib-based therapy for AL amyloidosis showing an overall response rate (ORR) of 65% with 49% of patients achieving a CR or VGPR^74^. Median overall survival was 72 months and not reached in those achieving a stringent CR (dflc < 10 mg/L). In using bortezomib-based therapy, one needs to be aware that response rate is lower in patients with t(11;14)^75^, a genetic abnormality seen in nearly 50% of patients with AL amyloidosis. The presence of t(11;14) should lead one to strongly consider stem cell transplantation over bortezomib, since this genetic abnormality does not have an unfavorable impact in transplanted patients. Predictors of early death after therapy initiation include the Mayo stage and greater than two organs involved. The value of addition of cyclophosphamide to bortezomib is debatable with some data suggesting limited additional benefit^76^.

#### Daratumumab

Daratumumab, a monoclonal antibody to CD38, approved for the treatment of relapsed multiple myeloma as a single agent as well as in combination with lenalidomide or bortezomib, clearly shows activity in the treatment of patients with AL amyloidosis^77^ and appears to have a low-toxicity profile. Early reports suggested a high response rate (76%) in heavily pretreated patients^78^. These data were confirmed by a number of other retrospective studies^79–81^. Two prospective phase II studies of daratumumab in combination with dexamethasone for relapsed AL amyloidosis have been with encouraging results. A multicenter French study of 40 patients receiving 6 months of daratumumab therapy showed an ORR of 70% with a median time to response of 1 week^82^. Similarly a phase II study conducted by the Boston group showed that daratumumab and dexamethasone (planned therapy for 2 years) led to a high ORR of 90% with a CR/VGPR rate of 86% and median time to response of 4 weeks^83^. The rates of organ response were 31 and 67% for renal response and 29 and 50% for cardiac response in the French and Boston studies respectively. Similar rates of organ response were reported, 52% renal and 55% cardiac, in a retrospective study of 72 patients receiving daratumumab and dexamethasone conducted at Stanford University^84^. Kimmich et al. reported 168 consecutive patients with relapsed AL amyloidosis treated with daratumumab, 62 of whom received this in combination with bortezomib and dexamethasone showing that this combination was well tolerated and efficacious in this population^85^. The early reports of success of daratumumab in AL amyloidosis led to the phase III ANDROMEDA study of VCd with or without daratumumab as upfront therapy for newly diagnosed AL amyloidosis patients. Early data reported from the safety run that included 28 patients showed an ORR of 96% and a CR rate of 54%^86^. The study reported interim analysis showing a CR rate of 53% for Dara-VCd and 18% for VCd (odds ratio, 5.1; 95% CI, 3.2–8.2; *P* < 0.0001) and better organ responses with the daratumumab containing regimen, cardiac response rate was 42% for Dara-VCd and 22% for VCd (*P* = 0.0029); 6-month-renal response rate was 54% and 27%, respectively (*P* < 0.0001)^87^. It was based on these impressive data that the FDA granted accelerated approval in January of 2021 for daratumumab in combination with bortezomib, cyclophosphamide and dexamethasone for newly diagnosed AL amyloidosis. This is the first FDA approval of an agent specifically for the treatment of AL amyloidosis and represents a landmark moment, suggesting that daratumumab will be a mainstay of treatment for AL amyloidosis in the future. Isatuximab is another monoclonal antibody to CD38 that has shown efficacy in myeloma and is now being studied in the treatment of AL amyloidosis. The SWOG S1702 study is a phase II clinical trial assessing the efficacy of Isatuximab in patients with previously treated AL amyloidosis (NCT03499808). Preliminary data on 36 patients recruited to the study were presented at the 62nd American Society of Hematology meeting showing an ORR of 77% and a median time to response of 1.1 months.

### Other agents

Because of the high prevalence of t(11;14) in AL amyloidosis patients, Venetoclax^88^, which has activity in multiple myeloma, particularly in those with the t(11;14), would be a natural candidate for the treatment of AL amyloidosis. A number of case reports showed efficacy in patients with AL amyloidosis^89–92^. A case series of 12 patients treated at Mayo Clinic showed hematologic response in 7 out of 8 evaluable patients, ORR 88%^93^. However given concerns regarding infection and mortality when used in combination with bortezomib and dexamethasone for myeloma in the phase III BELLINI study, use of this agent in AL amyloidosis patients who often have organ dysfunction needs to be carefully considered. A proposed phase I clinical trial is currently suspended (NCT03000660).

Carfilzomib, the second-generation proteasome inhibitor, has been tested^94^. A high incidence of cardiac involvement with AL amyloid makes it a challenging agent to use. Traditional pre- and post-hydration can aggravate patients predisposed to congestive heart failure. Carfilzomib is associated with cardiotoxicity in nearly 10% of patients. A review of Medicare admissions showed that carfilzomib-treated patients had a higher risk of hospitalization^95^. Hematologic responses have been reported in a small cohort of AL amyloidosis patients treated with upfront carfilzomib based therapy^96^. However its potential cardiotoxicity may be a barrier for wider implementation of this agent. Ixazomib has been shown to have a manageable toxicity profile and efficacious in patients with AL amyloidosis^97,98^. A phase 3 trial of ixazomib-dexamethasone versus physician-selected standard of care (TOURMALINE-AL1) was discontinued after it failed to show a significant improvement in hematologic response over physician choice (NCT01864018).

Bendamustine is an agent that is commonly used in non-Hodgkin lymphoma and has shown efficacy in myeloma. In a retrospective study of 122 patients with AL amyloidosis the combination of bendamustine with oral prednisolone led to an ORR of 35% with response rates higher in patients with IgM AL amyloidosis (58 vs. 28%). The toxicity profile was manageable and consistent with the use of bendamustine in other hematological malignancies. A prospective phase II study examined efficacy of bendamustine in combination with dexamethasone for relapsed AL amyloidosis and showed an ORR of 57% and organ response rate of 29%^99^. Grade 3-4 adverse events were seen in 65% and most commonly included myelosuppression, fatigue, and nausea.

Doxycycline has been used in patients with both AL^100^ and TTR^101–103^ amyloidosis with cardiac involvement. In vitro, doxycycline appears to disaggregate formed fibrils^104^. A trial from Mayo Clinic demonstrated that patients who achieved a hematologic response to stem cell transplant had a significantly longer overall survival post stem cell transplantation when given doxycycline compared to those receiving penicillin^105^. In a second study, which was case control, 26 patients receiving doxycycline were matched to 50 controls. The response rate was significantly higher in the doxycycline compared to controls, and the 12-month survival was 84 vs. 58%. Although there is no high-quality evidence and it has not been validated in a prospective randomized trial, doxycycline is a consideration if no other therapies are feasible. Preclinical studies suggest it may modulate amyloidogenic light chain mediated proteotoxicity in cardiac myocytes and studies using adjuvant doxycycline as part of therapy for AL amyloidosis have shown that this agent can be safely added to current treatment regimens^106,107^.

### Monoclonal antibodies to dissolve amyloid

Although chemotherapy can effectively reduce the light chain burden and disrupt further deposition in AL amyloidosis, it does nothing for resident amyloid in tissues. Although a number of agents in this setting have been studied over the recent years, only one is currently being evalusted in later stage clinical trials. The murine monoclonal antibody, 11-1F4, recognizes an amyloid-associated conformational epitope^108^. In a phase Ia/Ib study of 27 patients, 24 were evaluable for organ response and 12 of 18 (67%) patients with renal and/or cardiac involvement achieved a response. No toxicity >grade 3 was recognized^109–111^. This agent is being studied further in phase II and III clinical trials. Amyloid fibril targeted therapy with monoclonal antibodies is promising for the management of all forms of amyloidosis. Dissolution of amyloid fibrils may improve organ function.

### Organ transplantation

In AL amyloidosis, selected patients may successfully undergo renal or cardiac transplantation to assist with organ recovery. For patients that have single-organ involvement and control of the plasma cell proliferative process, organ transplantation may be considered. Stem cell transplantation can be safely performed in patients with dialysis-dependent^112^ renal failure^113^. Failure to achieve a complete response is no longer considered a contraindication to organ transplantation because of the increased availability of therapeutic options and direct organ donor programs. Once the patient has an established complete response, consideration of renal transplantation may be undertaken. Cardiac transplantation has also been performed in patients with AL amyloidosis^114^. Patients with advanced cardiac disease are unlikely to tolerate therapy well and achieve deep responses prior to consideration of organ transplantation. There have been cases of patients receiving cardiac allografting first, followed by chemotherapy or autologous stem cell transplantation to achieve remission of the AL amyloidosis^115–117^. Long-term survivorship has been reported in highly selected patients who fulfill the criteria of deep hematologic response and single-organ involvement^118^. Lenalidomide therapy is best avoided in organ transplant recipients that are considered for post-organ transplant chemotherapy^119^.

## Conclusion

Physician alertness and suspicion to the possibility of amyloidosis is a critical first step in diagnosing AL amyloidosis. This would allow earlier diagnosis, with less organ dysfunction, a setting in which treatments are likely to be most efficacious. Those diagnosed with advanced cardiac disease are unlikely to benefit from therapy and have a high mortality in the first few months of diagnosis. Once AL amyloidosis is suspected, the diagnosis can usually be made noninvasively, and organ biopsy is not generally required. Mass spectroscopic analysis should be standard for all newly diagnosed patients with amyloidosis to ensure correct classification of the protein subunit, and where unavailable immunohistochemical techniques may be utilized. All patients with light chain amyloidosis need cardiac biomarkers, free light chain measurements, and a bone marrow, with a thorough cardiac evaluation. The treatment of choice remains chemotherapy directed at the plasma cell clone producing the amyloidogenic light chain. In select cases stem cell transplantation may be considered, however only a minority of patients are eligible for this treatment and with the availability of newer agents that achieve deep responses, the utility of stem cell transplantation is likely to be questioned moving forward. For patients that are not transplant eligible but able to tolerate therapy, the standard of care would be combination therapy with daratumumab, bortezomib, cyclophosphamide, and dexamethasone based on the recent FDA approval of this combination for AL amyloidosis. Where daratumumab is unavailable, bortezomib-based regimens are preferred. For very frail patients, all oral therapy with melphalan and dexamethasone is appropriate. Second-line therapy can include immunomodulatory-based therapies, and other agents used in multiple myeloma, although data with use of these agents in this setting are sparse. If daratumumab has not been used upfront, we recommend using this agent at relapse with data showing very high response rates in this setting. Anti-amyloid antibodies are likely to have a potential role in the future management of these patients.
